# Nonlinear variation in clinging performance with surface roughness in geckos

**DOI:** 10.1002/ece3.6090

**Published:** 2020-02-22

**Authors:** Rishab Pillai, Eric Nordberg, Jendrian Riedel, Lin Schwarzkopf

**Affiliations:** ^1^ College of Science and Engineering James Cook University Townsville QLD Australia

**Keywords:** adaptation, adhesion biomechanics, ecomechanics, gekkota, physiology, zoology

## Abstract

Understanding the challenges faced by organisms moving within their environment is essential to comprehending the evolution of locomotor morphology and habitat use. Geckos have developed adhesive toe pads that enable exploitation of a wide range of microhabitats. These toe pads, and their adhesive mechanisms, have typically been studied using a range of artificial substrates, usually significantly smoother than those available in nature. Although these studies have been fundamental in understanding the mechanisms of attachment in geckos, it is unclear whether gecko attachment simply gradually declines with increased roughness as some researchers have suggested, or whether the interaction between the gekkotan adhesive system and surface roughness produces nonlinear relationships. To understand ecological challenges faced in their natural habitats, it is essential to use test surfaces that are more like surfaces used by geckos in nature. We tested gecko shear force (i.e., frictional force) generation as a measure of clinging performance on three artificial substrates. We selected substrates that exhibit microtopographies with peak‐to‐valley heights similar to those of substrates used in nature, to investigate performance on a range of smooth surfaces (glass), and fine‐grained (fine sandpaper) to rough (coarse sandpaper). We found that shear force did not decline monotonically with roughness, but varied nonlinearly among substrates. Clinging performance was greater on glass and coarse sandpaper than on fine sandpaper, and clinging performance was not significantly different between glass and coarse sandpaper. Our results demonstrate that performance on different substrates varies, probably depending on the underlying mechanisms of the adhesive apparatus in geckos.

## Graphical abstract

*Oedura coggeri* and *Pseudothecadactylus australis* occupy saxicolous and arboreal microhabitats. We tested their clinging ability on glass and other artificial substrates that exhibited similar peak‐to‐valley heights to substrates they use in nature to examine whether shear force declines monotonically with increasing peak‐to‐valley heights. We found that shear force did not decline monotonically with increasing peak‐to‐valley height.

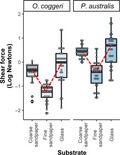

## 1 INTRODUCTION

An animal's fitness is strongly influenced by its locomotor ability, which is fundamental for successful prey capture and predator avoidance (Alexander, 2003). Successful locomotion in particular habitats is dependent on morphology, physiology, and habitat structure and is constrained by evolutionary history (Schriefer & Hale, 2004; Zani, 2000). Natural selection favors traits that optimize locomotor performance in various habitats, and variation in physiological and morphological characters may, in turn, increase performance in certain habitats (Kohlsdorf et al., 2004). Therefore, studies of ecological morphology and evolution often link morphology, performance, and ecology to suggest adaptation (Hagey, Puthoff, Crandell, Autumn, & Harmon, 2016; Wainwright & Reilly, 1994).

The ability to climb is widespread in the animal kingdom (Labonte & Federle, 2015). Adhesive toe pads evolved in many taxa as an adaptation to enhance clinging ability. These structures have independently evolved in multiple lineages such as lizards (Irschick et al., 1996; Russell, 2002), tree frogs (Hanna, Jon, & Barnes, 1991; Langowski, Dodou, Kamperman, & Leeuwen, 2018), arachnids (Niederegger & Gorb, 2006; Wolff & Gorb, 2016), and many insect orders (Bullock & Federle, 2011). The mechanisms of adhesion vary among taxa, however. Tree frogs use a combination of wet and dry adhesion (Labonte & Federle, 2015; Langowski et al., 2018), whereas lizards, insects, and arachnids have evolved a hierarchical adhesive system using van der Waals forces, although they act at different scales in different taxa (Labonte et al., 2016).

Subdigital adhesive toe pads in geckos represent a classic example of the evolution of locomotory traits that have evolved independently, on multiple occasions (Gamble, Greenbaum, Jackman, Russell, & Bauer, 2012, 2017; Irschick et al., 1996; Russell & Gamble, 2019), and enabled the exploitation of several habitat types. In geckos, subdigital pads consist of laterally expanded scales (called lamellae) covered with modified scale derivatives in the form of stalks termed setae (Maderson, 1964; Russell, 2002). Fields of microfibrillar setae adhere to contacted surfaces through van der Waals forces (Autumn, Dittmore, Santos, Spenko, & Cutkosky, 2006; Autumn et al., 2000; Tian et al., 2006). The ability to cling to substrates by means of subdigital pads has long been a topic of research (Collette, 1962; Delannoy, 2006; Elstrott & Irschick, 2004; Ernst & Ruibal, 1966; Gamble et al., 2012; Hagey, Puthoff, Holbrook, Harmon, & Autumn, 2014; Ruibal & Ernst, 1965), and several studies have aimed to determine factors that allow geckos to adhere to and detach from the substrates they move across, examining the locomotory substrate characteristics (Gillies et al., 2014; Meine, Kloss, Schneider, & Spaltmann, 2004; Persson & Gorb, 2003; Pugno & Lepore, 2008; Spolenak, Gorb, Gao, & Arzt, 2005), the mechanisms of adhesion (Autumn et al., 2002; Autumn, Niewiarowski, & Puthoff, 2014; Gao, Wang, Yao, Gorb, & Arzt, 2005; Irschick, Herrel, & Vanhooydonck, 2006; Mahendra, 1941; Tian et al., 2006), and variation in adhesion among species (Bergmann & Irschick, 2005; Garner, Stark, Thomas, & Niewiarowski, 2017; Hagey et al., 2014, 2017; Irschick et al., 1996; Stark, Klittich, Sitti, Niewiarowski, & Dhinojwala, 2016; Stark et al., 2015).

The gekkotan adhesive system has evolved to enable the exploitation of inclined and inverted surfaces on rocks, or vegetation, with recent expansions onto man‐made structures by some species (Glossip & Losos, 1997; Gamble et al., 2017; Hagey et al., 2017; Ruibal & Ernst, 1965). The mechanism and dynamics of adhesion, however, have almost exclusively been examined using a variety of smooth (Autumn et al., 2000; Gillies & Fearing, 2014; Irschick et al., 1996; Peressadko & Gorb, 2004; Russell & Johnson, 2007; Stewart & Higham, 2014) and very fine‐grained man‐made surfaces (i.e., glass, Teflon, variations of polyethylene, polyvinyl chloride, aluminum bonding wire, acrylic, and acetate sheets; Campolo, Jones, & Fearing, 2003; Gillies & Fearing, 2014; Huber, Gorb, Hosoda, Spolenak, & Arzt, 2007; Meine et al., 2004; Persson, 2003; Persson & Gorb, 2003; Pugno & Lepore, 2008; Vanhooydonck, Andronescu, Herrel, & Irschick, 2005; Winchell, Reynolds, Prado‐Irwin, Puente‐Rolón, & Revell, 2016), most of them not encountered by geckos under natural conditions. Such research has revealed that geckos perform better on substrates that are smooth, clean, and have uniform surface chemistry (Stark et al., 2015), apparently because these substrates provide a greater surface area with which setae can make contact (Russell & Johnson, 2007; Vanhooydonck et al., 2005).

Natural substrates are usually structurally and chemically substantially different from those used in laboratories (Russell & Johnson, 2007, 2014; Stark et al., 2015). A few recent studies have examined the surface topography of natural substrates and how it affects adhesion in geckos, highlighting the unpredictability (i.e., nonuniform amplitude and wavelengths of asperities creating varying undulance) of natural substrates, especially in comparison with artificial substrates previously used in gecko adhesion studies (Cole, Jones, & Harris, 2005; Naylor & Higham, 2019; Russell & Johnson, 2014; Vanhooydonck et al., 2005). Other studies have also stressed the importance of using ecologically relevant substrates to better understand performance in insects (Bullock & Federle, 2011), tree frogs (Langowski et al., 2019), and geckos (Hagey et al., 2014; Higham, Russell, Niewiarowski, Wright, & Speck, 2019; Niewiarowski, Stark, & Dhinojwala, 2016; Peattie, 2007). Most recently, Higham et al. (2019) summarized the importance, methods, and reasons for including ecological parameters like surface characteristics in gecko adhesion studies.

When setal fields are first deployed, spatulae make direct contact with the surface microtopography, and they go through a proximal pull, undergoing a preloading phase. This enables the generation of shear forces and increases the overall strength of the bond (Autumn, 2007; Autumn et al., 2000; Russell & Johnson, 2007). Hence, substrate surface microtopography has a major influence on the area available for attachment from a single spatula to the whole setal field and significantly influences the magnitude of force generated by the adhesive apparatus (Russell & Johnson, 2007). The peak‐to‐valley heights of the surface topology are one way to estimate roughness and therefore are also one way to assess the area available for setal contact at different microtopographies. Investigating the performance of geckos on surfaces with specific kinds of micro‐ and nanotopography is an important element of understanding adhesion in nature (Gamble et al., 2012; Russell & Johnson, 2007, 2014). Although studies on smooth artificial surfaces have been important for unraveling the physical principles behind gecko adhesion, it is not clear if such studies can be used to estimate performance, or relative performance, of different species of geckos on rougher or nonuniform surfaces, such as those they encounter in their natural environment.

**Figure 1:**
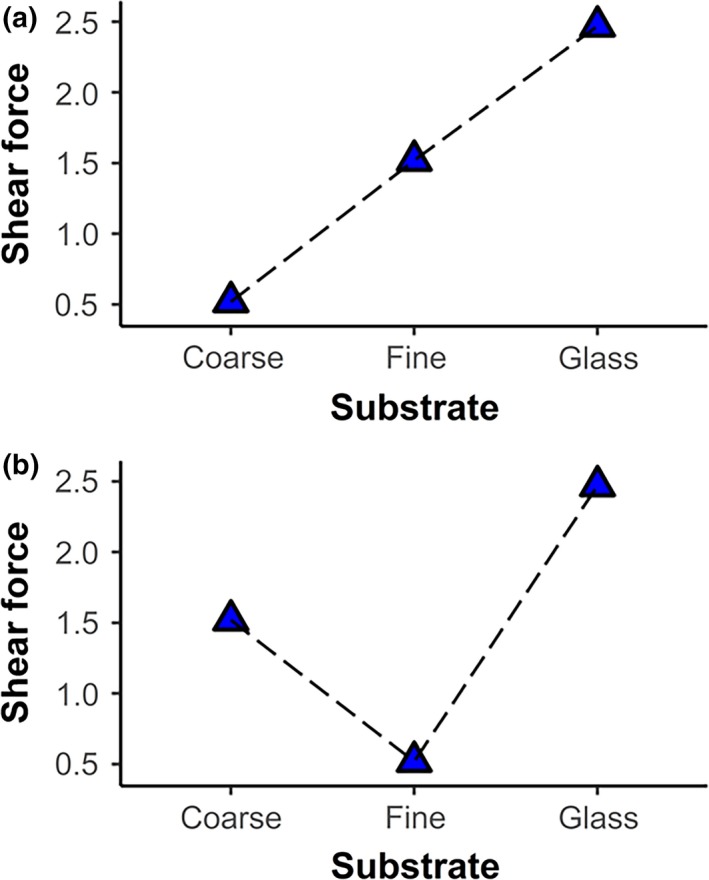
Conceptual model in which substrates are ordered by decreasing roughness (coarse sandpaper, fine sandpaper, and glass), suggesting (a) declining shear force with increasing roughness or (b) a nonlinear performance curve in relation to roughness. Points are joined to illustrate the expected shape of trends

Based on mechanisms predicted from observing gecko adhesion on artificial surfaces that are uniform and allow a very high proportion (nearing 100%, Russell & Johnson, 2007) of setae to make contact, we might expect a consistent decline in gecko attachment force with increasing roughness, presumably as setal fields find less purchase on uneven surfaces (Cole et al., 2005; Fuller & Tabor, 1975; Vanhooydonck et al., 2005; Figure 1a). Researchers have, however, found that setal fields can accommodate rougher surfaces, even though they are thought to have evolved for adhering to smooth substrates (e.g., *Rhoptropus* cf. *biporosus*; Russell & Johnson, 2014). In addition, recent studies have highlighted a multifunctional and synergistic relationship between claws and toe pads in geckos. Rough substrates that may provide limited surface area for setal attachment do allow mechanical purchase for claws. When substrates permit attachment of both claws and toe pads, that may increase clinging performance, even though there is limited surface area available for the setal fields by themselves. On the other hand, certain fine‐grained substrates do not permit secure attachment of claws or setal fields, leading to diminished clinging performance (Naylor & Higham, 2019). These combined processes may lead to a trend in which smooth substrates (permitting maximal engagement of setal fields) allow generation of great clinging performance, whereas, on certain coarse substrates, an intermediate proportion of the setal field can engage in conjunction with mechanical interlocking of claws. Further, the lowest performance presumably occurs on substrates of intermediate roughness, which provide poor purchase for both claws and setal fields (Figure 1b). Thus, surfaces with intermediate roughness may permit only partial contact, producing a nonlinear performance curve, if performance is plotted against peak‐to‐valley height, or roughness (Huber et al., 2007). In addition, some studies at very small scales suggest that surfaces with very low and quite high levels of roughness will permit increased contact between spatulae and the surface compared to surfaces with intermediate roughness (Huber et al., 2007), which would also give rise to a nonlinear graph of shear forces in relation to roughness.

Thus, we suggest there are multiple ways in which the adhesive apparatus of geckos could interact with substrates, which may give rise to different relationships between substrate roughness and shear forces generated. We predicted one of two possible trends in gecko attachment when examined on substrates with varying roughness (glass, fine sandpaper, and coarse sandpaper). (a) Performance might decline monotonically with increasing roughness (Figure 1a), or (b) performance might be lowest on surfaces with intermediate roughness forming a nonlinear trajectory (Figure 1b). We quantified shear forces produced by two gecko species with different morphology, body size, and habitats, along a roughness gradient. We aimed to investigate the shape of the response, as shear force generated versus peak‐to‐valley height of each surface.

## 2 METHODS

### 2.1 Study species

This study was conducted between August 2017 and December 2018. Two Diplodactylid gecko species (the northern spotted velvet gecko, *Oedura coggeri*, and the giant tree gecko, *Pseudothecadactylus australis*) were used to determine whether clinging ability imparted by geckos would decline monotonically with roughness or vary nonlinearly across substrates. Ten adult individuals (three males and seven females) of *O. coggeri*, a saxicolous species, were collected exclusively from rocky microhabitats around Paluma Range National Park, Queensland, Australia (GPS coordinates: −18.982772, 146.038974; datum = WGS84; 10 km radius), and housed at the James Cook University, Townsville Campus. Similarly, ten adult individuals (six males and four females) of *P. australis*, an arboreal species, were collected from tree bark and bamboo in Iron Range National Park, Queensland, Australia (GPS coordinates: −18.054768, 143.322002; 10 km radius), and were tested at a field station prior to release at their site of capture.

### 2.2 Ecological relevance of substrates

**Figure 2:**
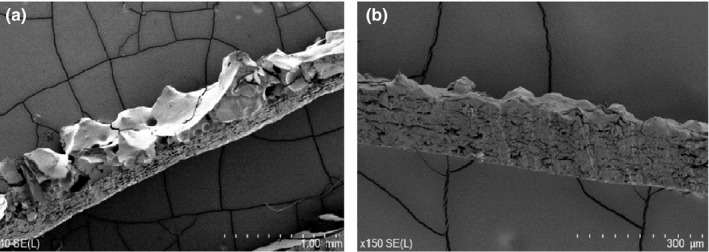
Lateral view of surface microtopography of sandpaper with peak‐to‐valley heights similar to natural substrates used to measure clinging ability in our study. (a) Cross section of coarse sandpaper (P40; 40× magnification); (b) cross section of fine sandpaper (P400; 150× magnification)

To select test substrates offering similar ecological challenges (at least in terms of peak‐to‐valley heights) to those faced by *O. coggeri* and *P. australis* in nature, we measured the peak‐to‐valley heights of natural substrates used by geckos (rock, tree bark, and bamboo samples collected at gecko capture sites). To quantify gecko clinging ability on surfaces at least partially representative of natural surfaces, we used coarse (P40) and fine (P400) sandpaper with similar peak‐to‐valley heights as test surfaces in this study (Figure 2). Additionally, glass was used as a test substrate as it is a smooth substrate, commonly used in gecko performance studies. Average peak‐to‐valley heights were measured using a surface profile gauge (Landtek Srt‐6223 Surface Profile Gauge, accuracy: ±5 µm; resolution: 0.1 µm/1 µm; range: 0–800 µm). Peak‐to‐valley heights were measured at 10 random points, within 10 cm of each other, in the laboratory for coarse and fine sandpaper, and from collected samples of rocks used by *O. coggeri*. Bamboo and bark substrates used by *P. australis* were measured in the field, using similar methodology. The surface profile gauge was calibrated prior to each measure using supplied standard glass exhibiting peak‐to‐valley heights of 0 µm. Differences in mean peak‐to‐valley heights (μm) among the substrate types were quantified using a Kruskal–Wallis test followed by a pairwise Wilcoxon post hoc analysis.

### 2.3 Clinging ability

We used three artificial surfaces (instead of using the natural surfaces used by the geckos) to ensure that the roughness characteristics and surface chemistry of the rougher surfaces were uniform. This approach allowed meaningful comparisons between species and surfaces, while providing measurements on substrates with peak‐to‐valley heights similar to those of natural substrates.

Prior to recording clinging ability, mass was measured once for each individual, using a digital scale (resolution: 0.01 g). To measure the surface area of toe pads, the ventral aspect of the hands and feet of all individuals was photographed through glass against a uniform dark background with a scale in each image. Lightroom CC (Adobe Systems Incorporated, 2019) was used to adjust the contrast of images to ensure that the emphasis was on the toe pads only. The thresholding feature in ImageJ (version 1.52a; Gillies & Fearing, 2014; Schneider, Rasband, & Eliceiri, 2012) was then used to select these toe pads by saturation, as they contrasted highly with the rest of the image. Measurements were calibrated using the scale incorporated in every image. We calculated the attachment area for each gecko on all five toes on the right hand (manus) and right foot (pes) of all geckos and doubled these measures to calculate the total attachment area for each individual. Each toe was measured once.

**Figure 3:**
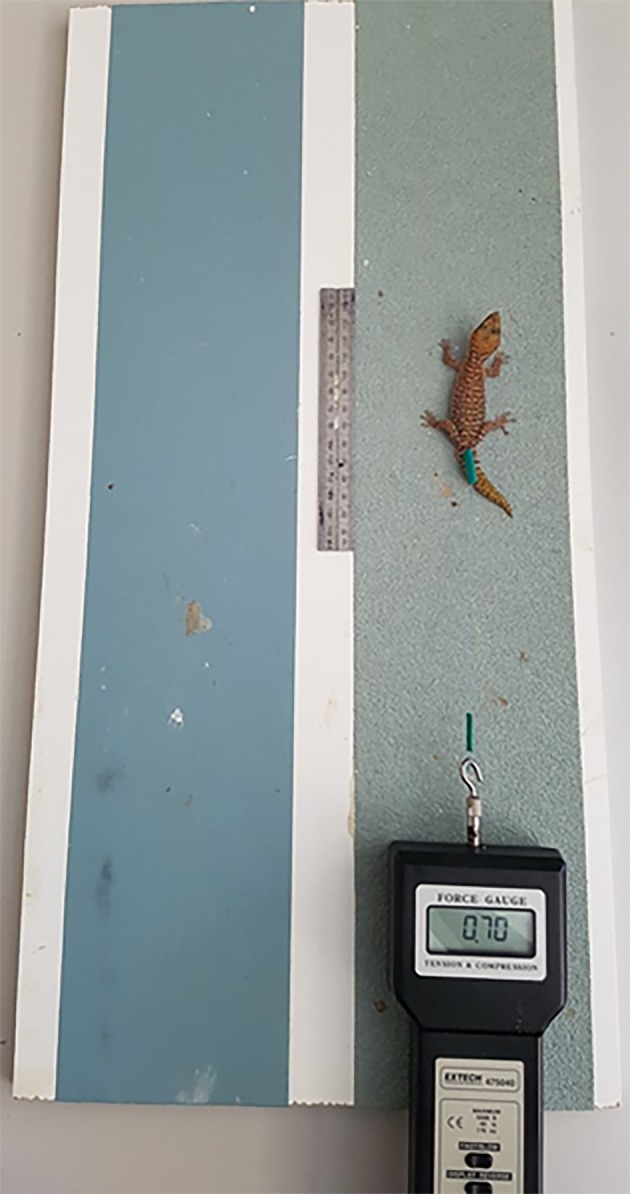
View from above of experimental setup used to measure shear force in the geckos *Pseudothecadactylus australis* and *Oedura coggeri*

To record the clinging ability of geckos when attached to a surface, we attached a force gauge (Extech 475040; resolution: 0.01 Newtons; maximum: 49 N ± 0.4% accuracy, Extech Equipment Pty Ltd) to the inguinal region of the gecko using a harness (Niewiarowski, Lopez, Ge, Hagan, & Dhinojwala, 2008) of fishing line (13.61 kg breaking strength; 0.5 mm diameter). Each gecko was permitted to take one step with each of its four feet on the testing substrate (P40, or P400 grit sandpaper, or glass), thereby ensuring that the natural adhesive system of the gecko was engaged (Collins, Russell, & Higham, 2015; Niewiarowski et al., 2008; Stark et al., 2015). Geckos were then pulled horizontally backward at an angle of 0° relative to the tabletop, using a constant velocity (~0.5 cm/s, calibrated using a 30‐cm ruler and stopwatch; Crandell, Herrel, Sasa, Losos, & Autumn, 2014; Irschick et al., 2005; Tulli, Abdala, & Cruz, 2011; Zani, 2000). Each individual lizard was tested three times on each surface (three measures per individual: Cole et al., 2005; McKnight et al., 2019; Tulli, Cruz, Herrel, Vanhooydonck, & Abdala, 2009) using all 10 individuals of each species. Order of testing on each surface type was randomized; therefore, we minimized the likelihood of damage caused to the adhesive apparatus by one substrate negatively influencing performance on another substrate. To reduce variation, the “toe pad engagement” of geckos was scored based on their level of attachment from a scale of 1–3 (highest to lowest attachment), and trials with scores higher than 3 were not included in this study (e.g., if a gecko tried to escape, or it did not appear to actively adhere the substrate, it received a higher score and the trial was excluded; Figure 3). Only one investigator (RP) conducted clinging ability trials to ensure consistency (Tulli et al., 2011). One measure of performance by *P. australis* on glass substrates was identified as an outlier (much >3 standard deviations from the mean) and was excluded from all further analysis.

Linear mixed‐effects models were used to quantify the differences in shear force exerted by both species on coarse and fine sandpaper and glass, in the R package *lme4* (Bates, Maechler, Bolker, & Walker, 2015). We constructed nine candidate models with three measures per individual on each substrate as our response variable. To account for intraindividual variation, all models included individual gecko IDs as random effects. Toe pad area is positively correlated with body size (mass; Collette, 1962; Irschick et al., 1996), and larger toe pads are more likely to have a larger setal field area, producing increased shear forces, which increase clinging ability (Irschick et al., 1996; Johnson & Russell, 2009; Russell & Johnson, 2014; Webster, Johnson, & Russell, 2009). Hence, the attachment force generated by the adhesive system on a substrate increases proportionally with an increase in toe pad area and with mass (Irschick et al., 1996). The species in our study had very different body sizes and toe pad areas (*O. coggeri*: mean mass = 7.48 g, whole animal mean toe pad area = 55.28 mm^2^; *P. australis*: mean mass = 20.21 g, whole animal mean toe pad area = 154.33 mm^2^); therefore, to account for the influence of mass and toe pad area on absolute force generated, we also included mass and toe pad area as fixed effects in all models, to control for their effects on clinging ability. Shear force, mass, and toe pad area were log‐transformed in all models (Table 1). Model selection was conducted using Akaike's information criterion (AIC) in the R package *AICcmodavg* (Mazerolle, 2019) to identify the model of best fit (ΔAIC < 2). We conducted post hoc analyses on the best‐fit model to identify differences within the fixed effects using the R package *emmeans* (Lenth, 2019). Results are reported as mean ± 1 standard error. All statistical analyses were conducted in R Studio (version 1.1.383, RStudio Team, 2016).

**Table 1:** Mixed‐effects models used to analyze shear forces exerted by the geckos *Pseudothecadactylus australis* and *Oedura coggeri*

Model number	Fixed effects	Random effects	Response variable
1	Substrate + log (toe pad area)	Individual gecko ID	Log (Shear force)
2	Substrate + log (mass)	Individual gecko ID	Log (Shear force)
3	Substrate + log (mass) + log (toe pad area)	Individual gecko ID	Log (Shear force)
4	Species + log (toe pad area)	Individual gecko ID	Log (Shear force)
5	Species + log (mass)	Individual gecko ID	Log (Shear force)
6	Species + log (toe pad area) + log (mass)	Individual gecko ID	Log (Shear force)
7	Substrate*Species + log (toe pad area)	Individual gecko ID	Log (Shear force)
8	Substrate*Species + log (mass)	Individual gecko ID	Log (Shear force)
9	Substrate*Species + log (toe pad area)	Individual gecko ID	Log (Shear force)

## 3 RESULTS

**Figure 4:**
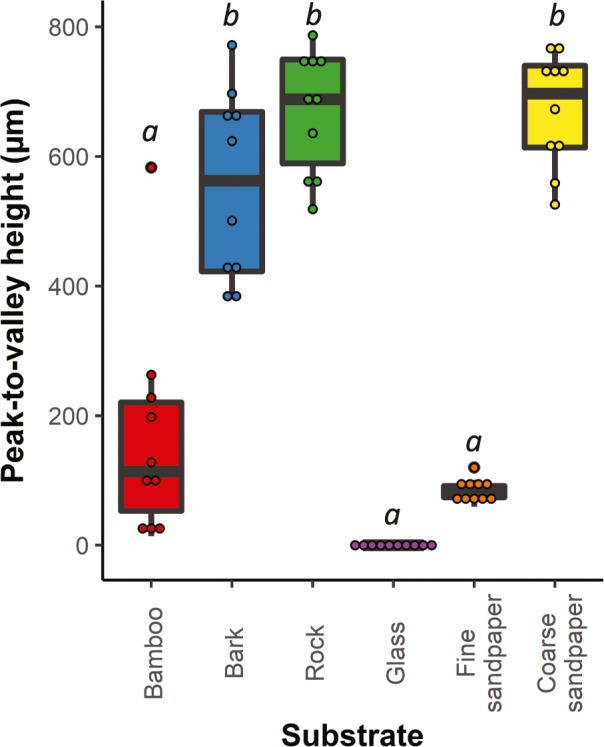
Peak‐to‐valley heights of substrates (µm). Substrates include natural surfaces used by *Pseudothecadactylus australis* and *Oedura coggeri* in nature (bark, rock, and bamboo) and test surfaces (coarse and fine sandpaper, and glass) used in this study. The artificial substrates were used to approximate the peak‐to‐valley heights of natural substrates used by geckos. Rocks used by the northern velvet geckos (*O. coggeri*) and bark used by giant tree geckos (*P. australis*) had similar average peak‐to‐valley height to coarse sandpaper. Bamboo, used by *P. australis*, had similar average peak‐to‐valley height to fine sandpaper. Significant differences between peak‐to‐valley heights of substrates are indicated by different italicized letters (Kruskal–Wallis test; pairwise Wilcoxon test; *α* = .05)

There was a significant difference in the mean peak‐to‐valley height of substrates (Kruskal–Wallis test: *p* < .001). The peak‐to‐valley heights of the coarse sandpaper (P40 grit) were not significantly different from those of rock or tree bark substrates used by *O. coggeri* and *P. australis* in nature (pairwise Wilcoxon test: tree bark: *p* = .14; rock: *p* = .12). The peak‐to‐valley heights of the coarse sandpaper (P40 grit) were significantly different from bamboo substrates used by *P. australis* (pairwise Wilcoxon test: *p* < .001). Peak‐to‐valley heights of bamboo substrates used by *P. australis* in nature were not significantly different from fine sandpaper (P400 grit; pairwise Wilcoxon test: *p* = .26). Glass had lower peak‐to‐valley heights than all other substrates (pairwise Wilcoxon test: bamboo: *p* < .001; bark: *p* < .001; rock: *p* < .001; coarse sandpaper: *p* < .001; Figure 4).

**Figure 5:**
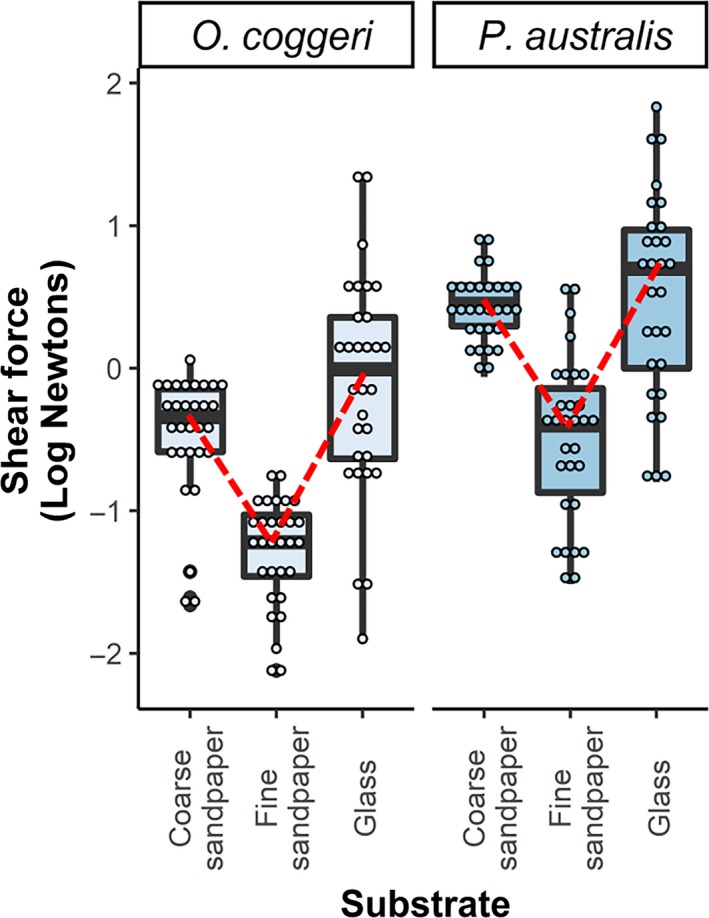
Clinging ability (log (Newtons)) of the northern spotted velvet gecko (*Oedura coggeri*) and giant tree gecko (*Pseudothecadactylus australis*) on glass, coarse sandpaper (P40 grit), and fine sandpaper (P400 grit). Both species performed significantly better on glass and coarse sandpaper than on fine sandpaper, producing a nonlinear trajectory, consistent with the prediction in Figure 1b (trend shape indicated with red dotted line)

The best model (ΔAIC < 2) predicting shear force exerted included substrate, mass, and toe pad area as fixed effects, with individual gecko IDs as random effects (conditional *R* ^2^ = .59, marginal *R* ^2^ = .64; Table 2 and 3). Shear force exerted by both species was significantly greater on glass (*O. coggeri*: 2.13 ± 0.64 N; post hoc comparison: *p* < .001; *P. australis*: 1.06 ± 0.20 N; post hoc comparison: *p* < .001) and coarse sandpaper (*O. coggeri*: 1.72 ± 0.51 N; post hoc comparison: *p* < .001; *P. australis*: 0.86 ± 0.16 N; post hoc comparison: *p* < .001) compared to fine sandpaper (*O. coggeri*: 0.72 ± 0.21 N; *P. australis*: 0.36 ± 0.07 N). Shear force exerted on glass and coarse sandpaper was not significantly different in either species (post hoc comparisons, *O. coggeri*: *p* = .18; *P. australis*: *p* = .18, Figure 5). Thus, shear forces did not decline in a linear fashion with roughness, as predicted in Figure 1a, but instead varied among substrates in a nonlinear trajectory, consistent with Figure 1b.

**Table 2:** Models included in selection using Akaike's information criterion, to analyze shear forces exerted by the geckos *Pseudothecadactylus australis* and *Oedura coggeri*

Fixed effects	ΔAIC	*df*	Weight	Residual deviance
Substrate + log (mass) + log (toe pad area)	0.0	7	0.449	269.1
Substrate*Species + log (mass)	1.2	10	0.247	264.3
Substrate + log (mass)	1.3	6	0.237	272.4
Substrate*Species + log (toe pad area)	3.8	9	0.066	269.0
Substrate + log (toe pad area)	18.7	6	<0.001	289.8
Substrate*Species + log (toe pad area)	19.0	9	<0.001	284.2
Species + log (mass)	109.5	5	<0.001	382.6
Species + log (toe pad area) + log (mass)	109.9	6	<0.001	381.1
Species + log (toe pad area)	117.2	5	<0.001	390.4

Note

The best model (ΔAIC < 2) included substrate, mass, and toe pad area as fixed effects. Models are arranged in increasing order of ΔAIC values.

Abbreviation: *df*, degrees of freedom.

**Table 3:** Fixed‐effects coefficient estimates of the linear mixed‐effects model for the differences in shear force on substrates with different peak‐to‐valley heights

	Estimate	*SE*	*df*	*t*	*p*
Intercept^a^	−2.37	0.37	173.13	−6.365	>.001
Fine sandpaper	−0.87	0.09	168.92	−9.609	>.001
Glass	0.21	0.09	168.99	2.344	>.050
log (mass)	0.54	0.12	135.02	4.69	>.001
log (toe pad area)	0.23	0.12	152.61	1.83	.060

Abbreviations: *df*, degrees of freedom; *p*, *p*‐value; *SE*, standard error; *t*, *t*‐statistic.

a Shear force on coarse sandpaper.

## 4 DISCUSSION

Both *P. australis* and *O. coggeri* exerted significantly higher shear forces on glass and coarse sandpaper than on fine sandpaper. Therefore, we did not observe a monotonic decline in performance with increasing peak‐to‐valley heights, which contrasts with findings of studies in which performance diminished considerably with increasing levels of roughness (Cole et al., 2005; Vanhooydonck et al., 2005). Shear force exerted on coarse substrates was not significantly different from that on glass in either species; thus, our results showed a nonlinear relationship between peak‐to‐valley heights and shear forces on the continuum of surfaces we used, consistent with studies by Huber et al. (2007; on a scale of single spatula), and Naylor and Higham (2019). Gecko adhesive systems have been well studied on a range of artificial substrates that have revealed the form and function of the adhesive apparatus in this taxon; however, our findings further highlight the need for gecko adhesion studies under more ecologically relevant conditions (Collins et al., 2015; Higham & Russell, 2010; Higham et al., 2019; Russell & Delaugerre, 2017). More comparative studies examining gecko attachment on different substrates are needed to elucidate the potentially context‐specific nature of gecko attachment.

The shear force that can be generated by geckos is thought to be impacted by surface topology because topology determines the area available for attachment at the scale of the setal fields and also the degree to which claws can be effective. Natural substrates have microtopographies that are unpredictable and nonuniform compared to glass and other artificially smooth substrates (Russell & Johnson, 2007, 2014), highlighting the importance of overall structural considerations of locomotory substrates in gecko adhesion studies (Higham et al., 2019). The peak‐to‐valley heights of the coarse sandpaper we used to measure gecko clinging ability were similar to those of the rock and bark microhabitats used by *O. coggeri* and *P. australis*, respectively. Additionally, the fine sandpaper used in our study was similar in peak‐to‐valley height to bamboo surfaces used by *P. australis* in nature. There are, however, a range of other characteristics of rough surfaces that may influence attachment, such as variation in amplitude, wavelength (Gillies et al., 2014), spacing (Zhou, Robinson, Steiner, & Federle, 2014), and microstructuring of surface asperities, which could affect conformity of the adhesive apparatus or the attachment of claws. Additionally, the chemistry of the surfaces could influence interaction strength (Prüm, Bohn, Seidel, Rubach, & Speck, 2013), although we controlled for surface chemistry on both our rough surfaces by using the same brand of sandpaper, instead of using natural substrates. More research is required to determine the importance of exact topography and chemistry in replicating characteristics of natural substrates and to address the challenges of describing and quantifying surface roughness (Higham et al., 2019; Persson, Tiwari, Valbahs, Tolpekina, & Persson, 2018). Future research should incorporate carefully described and quantified, realistic surfaces in laboratory studies of attachment (Higham et al., 2019; Langowski et al., 2018).

We found that shear forces exerted by both *P. australis* and *O. coggeri* were greater on glass compared to on fine sandpaper. The gekkotan adhesive system is often characterized as most efficient on smooth substrates (Russell, Baskerville, Gamble, & Higham, 2015). High performance on glass, observed in our study, was consistent with previous studies that have tested clinging ability on artificial smooth substrates (Autumn et al., 2006, 2000; Huber et al., 2007; Irschick et al., 1996; Mahendra, 1941; Naylor & Higham, 2019). Smoother surfaces provide an increased area onto which fields of setae can make simultaneous contact, and generate substantial force (Russell & Johnson, 2007). Both species exhibited their highest clinging ability on glass. Our findings were consistent with the findings of previous studies in which instantaneous acceleration (40 m/s^2^ on wood with 98% surface area available for attachment; Vanhooydonck et al., 2005) and maximum clinging ability (~2.5 N on acrylic with 0.0 root mean square height Sq [µm]; Naylor & Higham, 2019) were highest on substrates that provided high surface area for attachment.

In our study, shear forces exerted on coarse substrates were not significantly different from those on glass, showing that the gekkotan attachment system also attaches efficiently to rough substrates. The question remains, however, what is the source of this effective attachment? Studies examining attachment systems consisting of claws and adhesive hairs in geckos (Naylor & Higham, 2019) and other taxa (rove beetles: Betz, 2002; dock beetle: Bullock & Federle, 2011; leaf beetles: Voigt, Schweikart, Fery, & Gorb, 2012) have demonstrated that claws are a critical aspect of clinging in nature, and suggest that there may be a synergistic relationship between claws and setae. They propose that greater attachment is achieved on surface topographies onto which both components can attach (Song, Dai, Wang, Ji, & Gorb, 2016). In our study, the nonlinear relationship of adhesion with roughness may have occurred because setal fields could maximize contact on smooth surfaces compared to fine‐grained substrates. The lower generation of shear forces on fine‐grained substrates was possibly because the opportunity for mechanical interlocking of claws was reduced on the finer‐grained sandpaper. Fine‐grained substrates are less likely to permit claws to attach compared to coarse substrates, producing the lowest generation of shear forces on fine‐grained substrates in our study. On coarse surfaces, claws could mechanically interlock, compensating for the lack of effectiveness of setae on such surfaces and increasing overall shear forces. Other studies suggest that rough surfaces provide plenty of purchase for the setal system alone (Russell & Johnson, 2014). For example, the African geckos *Rhoptropus* cf. *biporosus* attached well to sandstone substrates, even though they lack tractive claws (Russell & Johnson, 2014). Additionally, Langowski et al. (2019) also report a similar trend in tree frogs, which lack claws entirely. Such observations suggest that the nonlinear performance graph we observed may not be driven solely by the relative role of claws in the adhesive apparatus of geckos. Experiments disabling setal fields or claws, while determining the role of the other part of the clinging apparatus on surfaces of various roughnesses, are required to further examine the hypotheses raised by these observations.

*Pseudothecadactylus australis* uses bamboo substrates in nature, but they exerted lower shear forces on fine sandpaper with peak‐to‐valley heights similar to bamboo substrates. Our field observations show that *P. australis* used bamboo substrates less often than tree bark (one observation on bamboo and 25 observations on tree bark). Possibly, bamboo substrates do not permit sufficient setal contact nor do they provide the undulance required for mechanical interlocking of claws, and so they are not preferred substrates for these geckos. Further studies should record microhabitat selection and investigate clinging ability in relation to preferred microhabitats.

Our results show that gecko clinging performance did not decline monotonically with increasing peak‐to‐valley heights of substrates. Instead, performance was lowest on the substrate with intermediate peak‐to‐valley heights and was similar on glass and coarse sandpaper. Our findings demonstrate that gecko attachment forces can be context‐dependent and provide a basis for further studies examining the role of substrate and the different elements (claws and setae) in gecko attachment. Further, our study showed: (a) complex mechanisms promoting gecko attachment on multiple substrates with different microtopography, and illustrated that geckos can cling well to rough substrates thought to offer limited accommodation for the adhesive apparatus of geckos (Naylor & Higham, 2019; Russell & Johnson, 2007, 2014); and (b) that measuring performance using substrates with ecologically relevant roughness enables the quantification of clinging ability within a range that is biologically and evolutionarily meaningful (Bartholomew, 2005; Hagey et al., 2014; Higham et al., 2019; Langowski et al., 2018; Niewiarowski, Stark, McClung, Chambers, & Sullivan, 2012; Peattie, 2007; Russell & Johnson, 2007, 2014).

## CONFLICT OF INTEREST

The authors have no competing interests.

## AUTHOR CONTRIBUTIONS

RP, EN, JR, and LS conceived the ideas and designed the methodology; RP collected the data; RP, EN, JR, and LS analyzed the data; and RP led the writing of the manuscript. All authors contributed critically to the drafts and gave final approval for publication.

## Data Availability

Ecological relevance of substrates and clinging ability data: Dryad https://doi.org/10.5061/dryad.9w0vt4bbd
